# A Case Report of Benign Pancreatic Hyperenzymemia (Gullo’s Syndrome)

**DOI:** 10.7759/cureus.8143

**Published:** 2020-05-15

**Authors:** Parth Mehta, Anil Kumar Reddy Reddivari

**Affiliations:** 1 Internal Medicine, University of Illinois College of Medicine, Peoria, USA; 2 Internal Medicine, Unity Point Health Methodist Hospital, Peoria, USA

**Keywords:** gullo's syndrome, hyperenzymemia, pancreas

## Abstract

Benign Pancreatic Hyperenzymemia or Gullo’s Syndrome is a rare syndrome that has been identified relatively recently and is characterized by abnormally elevated serum pancreatic enzymes in the absence of any clinical or pathological evidence of pancreatic disease. It is usually discovered incidentally, occurs sporadically or as a familial form and remains a diagnosis of exclusion. Both amylase and lipase are elevated but can return to normal levels temporarily. We present an interesting case of benign pancreatic hyperenzymemia. This case highlights the importance of identifying this condition to avoid unnecessary testing and reassuring the patient of its benign nature.

## Introduction

Pancreatic diseases are usually associated with an increase in the levels of the serum pancreatic enzymes. But few other non-pancreatic diseases have also been reported to have elevated levels of pancreatic enzymes, for example hypertriglyceridemia, macroamylasemia, duodenal ulcer, bowel obstruction, liver disease, malignancy, acute cholecystitis, etc [1]. Gullo described a new syndrome in 1996 which was characterized by persistently elevated levels of pancreatic enzymes in absence of clinical or pathological evidence of pancreatic disease and hence now known as benign pancreatic hyperenzymemia or Gullo’s syndrome [2]. 

## Case presentation

A 43-year-old female with a history of renal stones status post lithotripsy was admitted for abdominal pain, urinary frequency and high-grade fever with chills that were going on for five days. Vitals signs on initial presentation showed a temperature of 103.9 °F, pulse rate of 98/minute, blood pressure of 102/52 mm Hg and respiratory rate of 16/minute. Physical examination was remarkable for mild abdominal tenderness on deep palpation in mid to lower abdomen. The rest of the physical examination was essentially normal. 

Initial workup revealed hemoglobin of 13.1 g/dl, hematocrit 40.9%, white blood cell count of 17.9X10^3^/uL, and platelet count of 250X 10^3^/uL. Metabolic panel and hepatic function panel were completely unremarkable with normal creatinine and transaminases. Urinalysis showed many bacteria and 100+ white blood cells and was positive for leukocyte esterase and nitrites. Serum lipase and amylase level had been checked on presentation due to abdominal pain and came back elevated at 1142 U/L (Normal: 40-700 U/L) and 1024 U/L (Normal: 30-600 U/L) respectively. Lipase and amylase levels were monitored on a daily basis for 8 days and showed persistent elevation (Table 1). Isoamylase was not ordered due to the unavailability of the test at the facility. Abdominal pain resolved the next day. 

**Table 1:** Levels of serum lipase and amylase levels over a period of 8 days from the time of admission.

Day of Admission	Serum Lipase (U/L)	Serum Amylase (U/L)
Day 1	1142	1024
Day 2	1309	1123
Day 3	2812	2213
Day 4	2613	2478
Day 5	2308	1872
Day 6	1638	1549
Day 7	1287	1156
Day 8	944	688

**Figure 1:**
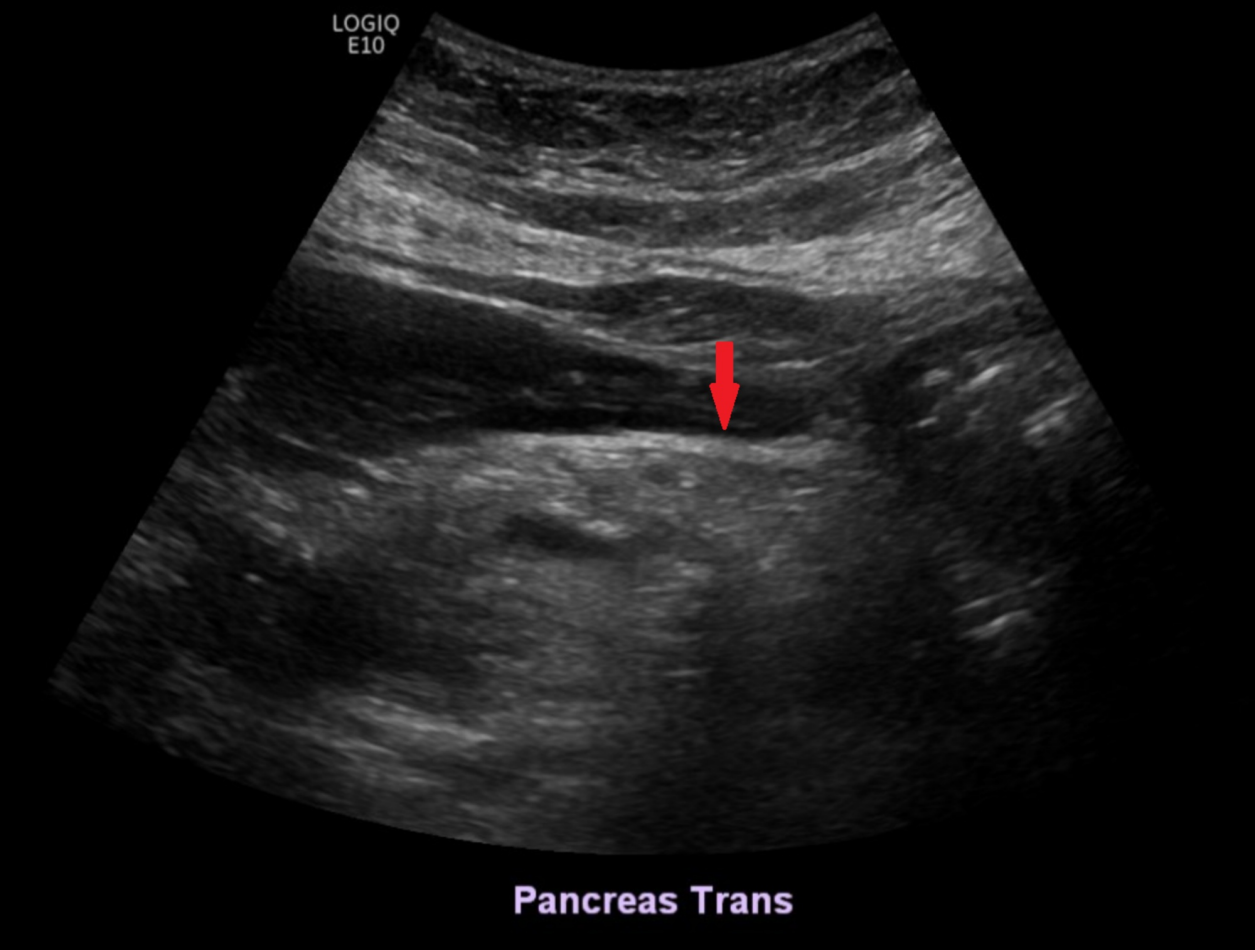
Ultrasound of the abdomen showing normal heterogeneous pancreas with hypo and hyperechogenicity of the parenchyma of the normal-sized pancreatic head and body, negative for parenchymal lesions or pancreatitis. The pancreatic tail was sub-optimally assessed due to overlying bowel gas.

**Figure 2:**
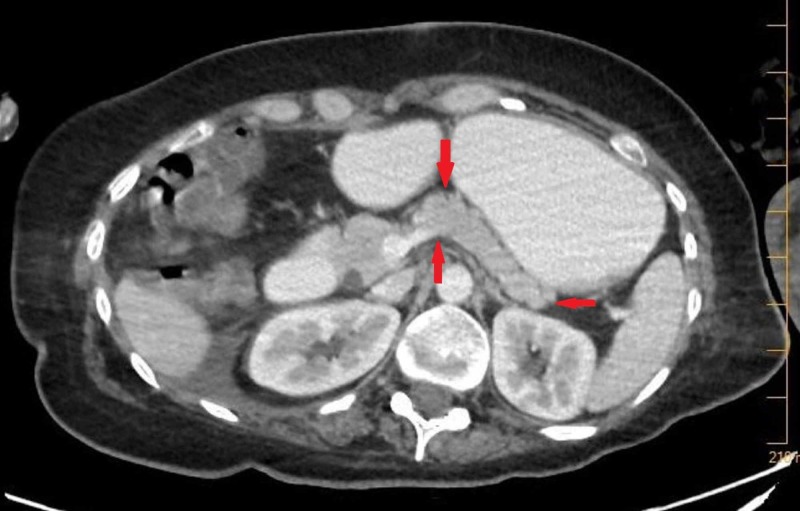
CT scan of the abdomen with contrast showing the normal pancreas without any pancreatitis or pancreatic lesions.

**Figure 3:**
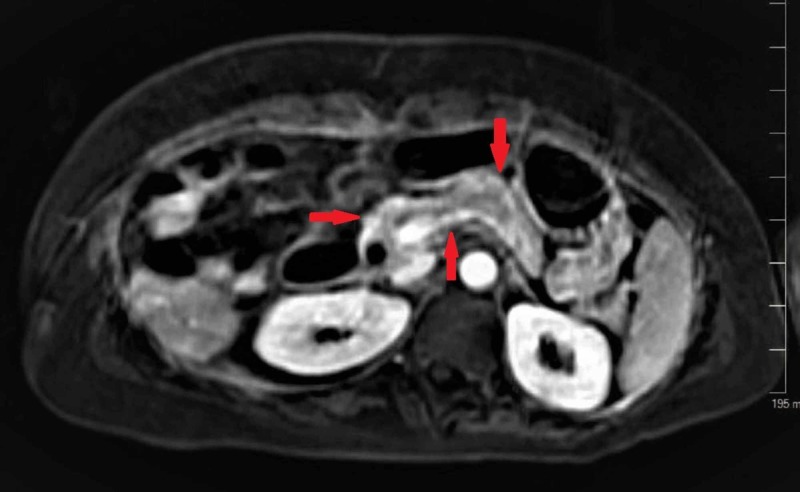
MRI of the abdomen with contrast showing the normal pancreas without any pancreatitis or pancreatic lesions.

Due to elevated pancreatic enzymes in the absence of any ongoing symptoms, further work to rule out any pancreatic etiology was done. The patient then underwent an ultrasound of the abdomen, CT scan of the abdomen with contrast and MRI of the abdomen with contrast. All three imaging studies were negative for any pancreatitis or pancreatic lesions and also did not show any other gastric, hepatic, biliary or small bowel abnormalities (figure 1-3).

Urine culture grew more than 100,00 colonies/ml E. coli. The patient was treated for sepsis secondary to UTI with appropriate antibiotics. Urinary frequency and high-grade fever with chills started improving and resolved 5 days after the patient was started on antibiotics. The patient was going to be discharged on the 5th day but her blood culture grew E.coli and she was kept for an additional two days. Repeat blood culture was done and remained negative. The patient was discharged on the 8th day of the admission.

She was followed up for eighteen months and remained completely asymptomatic without any recurrence of abdominal pain. Serum lipase and amylase levels were checked at twelve months and were found to be elevated at 832 U/L and 651 U/L respectively. At eighteen months, serum lipase and amylase levels were rechecked and were still found to be elevated at 768 U/L and 590 U/L respectively, leading to a diagnosis of Benign Pancreatic Hyperenzymemia. The patient was offered to undergo a repeat imaging study but she declined any further imaging.

## Discussion

Pancreatic disorders are usually associated with elevated pancreatic enzymes. However, in benign pancreatic hyperenzymemia, persistent elevation of pancreatic enzymes including lipase, amylase, isoamylase without clinical or pathologic evidence of pancreatic disease with a normal pancreas on imaging studies is observed. Enzyme levels remain persistently elevated but can transiently return to normal levels and at least one year must pass after detection of hyperenzymemia before labeling it as Gullo’s syndrome. This phenomenon was first described by the Italian gastroenterologist Dr. Lucio Gullo in 1996 where a healthy man with elevated serum pancreatic enzymes was admitted with a suspected pancreatic disease but all the imaging tests were normal. Initially, it was thought that persistent enzyme elevation could be due to chronic pancreatitis or pancreatic tumor, but no definite pathology was found. Dr. Gullo published several articles on it and hence it is known as Gullo’s syndrome [3]. 

Benign pancreatic hyperenzymemia can occur sporadically or in familial form. The ratio of affected men and women is 1.5:1 but it can occur in children too. As it has been detected in more than one member of the family, the genetic basis for this disease has also been hypothesized. However, the exact mechanism remains unclear. CFTR (cystic fibrosis transmembrane regulator) mutation has been observed in patients with Gullo’s syndrome. However, the frequency of this mutation is similar compared to the general population [4]. Some patients were evaluated by Dr. Gullo for PRSS1 mutation that is linked to hereditary pancreatitis and SPINK1 mutation that is linked to the pancreatitis of different etiologies. However, the PRSS1 mutation was not detected and the SPRINK1 mutation frequency was similar to the general population [5]. Relationship with celiac disease was studied as macroamylasemia is commonly associated with celiac disease where the chronic elevation of amylase levels is seen and lipase levels remain normal. However, in patients with Gullo’s syndrome, pancreatic enzymes did not normalize after the gluten-free diet. Gullo’s syndrome is also believed to be caused by a defect in the basolateral surface of the acinar cells causing an increase in the passage of enzymes into the blood or by the effect of secretin in the pancreatic duct of Wirsung. However, the study showed that change in the diameter of the duct was similar to the control group [6]. 

It is, however, an important point to remember that patients must be followed for a period of at least one year before labeling their pancreatic hyperenzymemia as benign because in 1-2% of cases of pancreatic cancer, asymptomatic pancreatic hyperenzymemia can be an early witnessed laboratory abnormality [7]. 

## Conclusions

Patients who have benign pancreatic hyperenzymemia or Gullo’s syndrome are not at high risk for developing pancreatitis. Since it is a benign syndrome, it is important to be aware of this condition to reassure patients to not worry and to avoid subjecting them to unnecessary testing and hospitalizations. However, it is also important to remember that patients must be followed for a period of at least one year before labelling their pancreatic hyperenzymemia as benign as in few cases of pancreatic cancer, asymptomatic pancreatic hyperenzymemia can be an early witnessed laboratory abnormality.
